# Primary cutaneous B‐cell lymphoma other than marginal zone: clinicopathologic analysis of 161 cases: Comparison with current classification and definition of prognostic markers

**DOI:** 10.1002/cam4.865

**Published:** 2016-09-26

**Authors:** Marco Lucioni, Emilio Berti, Luca Arcaini, Giorgio A. Croci, Aldo Maffi, Catherine Klersy, Gaia Goteri, Carlo Tomasini, Pietro Quaglino, Roberta Riboni, Mariarosa Arra, Elena Dallera, Vieri Grandi, Mauro Alaibac, Antonio Ramponi, Sara Rattotti, Maria Giuseppina Cabras, Silvia Franceschetti, Giulio Fraternali‐Orcioni, Nicola Zerbinati, Francesco Onida, Stefano Ascani, Maria Teresa Fierro, Serena Rupoli, Marcello Gambacorta, Pier Luigi Zinzani, Nicola Pimpinelli, Marco Santucci, Marco Paulli

**Affiliations:** ^1^ Unit of Anatomic PathologyDepartment of Molecular MedicineUniversity of Pavia and Fondazione IRCCS Policlinico San MatteoPaviaItaly; ^2^ Department of DermatologyFondazione IRCCS Ca’ Granda ‐ Ospedale Maggiore Policlinico and Università degli Studi di Milano‐BicoccaMilanItaly; ^3^ Section of Hematology‐OncologyDepartment of Molecular MedicineUniversity of PaviaPaviaItaly; ^4^ Scientific DirectionBiometry and StatisticsFondazione IRCCS Policlinico San MatteoPaviaItaly; ^5^ Pathologic Anatomy and HistopathologyDepartment of Biomedical Sciences and Public HealthPolytechnic University of Marche RegionUnited Ancona HospitalsTorretteAnconaItaly; ^6^ Pathology UnitDepartment of Laboratory MedicineAzienda Ospedaliera Città della Salute e della Scienza di TorinoTurinItaly; ^7^ Dermatologic ClinicDepartment of Medical SciencesUniversity of TurinTurinItaly; ^8^ Division of DermatologyUniversity of Florence Medical SchoolFlorenceItaly; ^9^ Dermatologic ClinicUniversity of PadovaPadovaItaly; ^10^ Division of PathologyAzienda Ospedaliero‐Universitaria Maggiore della CaritàNovaraItaly; ^11^ Department of Hematology and OncologyFondazione IRCCS Policlinico San MatteoPaviaItaly; ^12^ Division of HematologyOsp A BusincoCagliariItaly; ^13^ Division of HematologyDepartment of Translational MedicineAmedeo Avogadro University of Eastern PiedmontAzienda Ospedaliero‐Universitaria Maggiore della CaritàNovaraItaly; ^14^ Anatomic Pathology DivisionSan Martino University HospitalGenovaItaly; ^15^ Department of Surgical and Morphological SciencesFaculty of Medicine and SurgeryUniversity of InsubriaVareseItaly; ^16^ Hematology and Bone Marrow Transplantation CenterFondazione IRCCS Ca’ Granda Ospedale Maggiore Policlinico and University of MilanMilanoItaly; ^17^ Institute of PathologyOspedale S. Maria di Terni and University of PerugiaPerugiaItaly; ^18^ Clinic of HematologyUnited Ancona HospitalsTorretteAnconaItaly; ^19^ Synlab, ItaliaBresciaItaly; ^20^ Institute of Hematology “Seràgnoli”University of BolognaBolognaItaly; ^21^ Division of Pathological AnatomyDepartment of Surgery and Translational MedicineUniversity of Florence School of Human Health SciencesFirenzeItaly

**Keywords:** BCL2, cutaneous lymphoma, follicular lymphoma, large cell lymphoma, leg type

## Abstract

Categorization of primary cutaneous B‐cell lymphomas (PCBCL) other than marginal zone (MZL) represents a diagnostic challenge with relevant prognostic implications. The 2008 WHO lymphoma classification recognizes only primary cutaneous follicular center cell lymphoma (PCFCCL) and primary cutaneous diffuse large B‐cell lymphoma, leg type (PCDLBCL‐LT), whereas the previous 2005 WHO/EORTC classification also included an intermediate form, namely PCDLBCL, other. We conducted a retrospective, multicentric, consensus‐based revision of the clinicopathologic characteristics of 161 cases of PCBCL other than MZL. Upon the histologic features that are listed in the WHO classification, 96 cases were classified as PCFCCL and 25 as PCDLBCL‐LT; 40 further cases did not fit in the former subgroups in terms of cytology and/or architecture, thus were classified as PCDLBCL, not otherwise specified (PCDLBCL‐NOS). We assigned all the cases a histogenetic profile, based on the immunohistochemical detection of CD10, BCL6, and MUM1, and a “double hit score” upon positivity for BCL2 and MYC. PCDLBCL‐NOS had a clinical presentation more similar to PCFCCL, whereas the histology was more consistent with the picture of a diffuse large B‐cell lymphoma, as predominantly composed of centroblasts but with intermixed a reactive infiltrate of small lymphocytes. Its behavior was intermediate between the other two forms, particularly when considering only cases with a “non‐germinal B‐cell” profile, whereas “germinal center” cases resembled PCFCCL. Our data confirmed the aggressive behavior of PCDLBC‐LT, which often coexpressed MYC and BCL2. The impact of single factors on 5‐year survival was documented, particularly histogenetic profile in PCDLBCL and BCL2 translocation in PCFCCL. Our study confirms that a further group—PCDLBCL‐NOS—exists, which can be recognized through a careful combination of histopathologic criteria coupled with adequate clinical information.

## Introduction

The issue of classification of primary cutaneous B‐cell lymphomas (PCBCL) other than marginal zone lymphoma (MZL) has been matter of debate. The 2008 WHO Lymphoma Classification 1 recognizes two subtypes: primary cutaneous follicular center cell lymphoma (PCFCCL) and primary cutaneous diffuse large B‐cell lymphoma, leg type (PCDLBCL‐LT). PCFCCL is defined on the basis of cytological features (presence of centrocytes) irrespective of growth pattern, which may be variable from follicular to predominantly diffuse; in some case, mostly advanced tumors, the lymphoma infiltrate may contain a prevalence of large cells, a feature which seems not to affect prognosis. PCDLBCL‐LT designs all cutaneous B‐cell lymphomas with a diffuse pattern and composed of monotonous proliferation of centroblasts and immunoblasts, usually BCL2‐positive, irrespective of site of presentation. This two‐tiered distinction was validated by clinical studies 2, 3 and was partially supported by the identification of different molecular signatures and imbalances 4 in PCFCCL and PCDLBCL‐LT, the latter resembling the activated B‐cell type (ABC) of nodal DLBCL 5, 6.

In the previous WHO/EORTC classification (2005) 7, 8, the heading of cutaneous diffuse large B‐cell lymphomas comprised several variants, including PCDLBCL‐LT, cases with peculiar morphology (T‐cell/histiocyte rich, plasmablastic) as well as diffuse lymphomas of centroblastic‐like cells, intermingled with a mixed inflammatory infiltrate and with variable expression of BCL2, which are named primary cutaneous diffuse large B‐cell lymphoma, other (PCDLBCL‐O). PCDLBCL‐O basically represents a morphological variant lacking the typical features of PCDLBCL‐LT neither conforming to the definition of PCFCCL, whereas on the clinical ground, its behavior seems at least to partially overlap the indolent course of PCFCCL. In fact, the present WHO lymphoma classification overcame the previous WHO/EORTC and included at least a part of PCDLBCL‐O within the spectrum of PCFCCL.

In spite of the advances in the classification, the identification of this putative variant remains not trivial, since it might harbor significant prognostic and therapeutic implications. While the 5‐year disease‐specific survival in PCDLBCL‐LT is 41%, PCFCCL carries an excellent prognosis, with a 95% 5‐year survival 1 even in cases featuring a predominance of large cells, which may benefit from a conservative therapeutic approach. Since only few studies focused on such issue and indeed no conclusive data are available on large series 9, 10, question still remains whether such group of PCBCL with borderline features between PCFCCL and PCDLBCL‐LT could define a further distinct category.

To clarify the existence of an additional clinicopathologic subset of PCLBCL, we retrospectively analyzed a large multicentric series of PCBCL other than MZL and tested the prognostic relevance of several factors, including cytomorphologic features, histogenetic profiles, and BCL2 status 11.

## Methods

### Selection of patients

This multicentric study retrospectively analyzed the clinicopathologic features of a series of 197 PCBCL other than MZL, diagnosed between 1993 and 2010 at 10 centers referring to the “Gruppo Italiano di studio dei Linfomi Cutanei (G.I.L.C.)” of the “Fondazione Italiana Linfomi (F.I.L.).” Approval for this study was obtained from the local institutional ethical committee. Data management was made according to the Helsinki Declaration of 1975, revised in 1983 and 2000.

Inclusion criteria were as follows: (1) primary cutaneous disease, documented through comprehensive staging, and no extracutaneous spread for at least 6 months after diagnosis; (2) availability of representative formalin‐fixed, paraffin‐embedded (FFPE) lesional blocks; and (3) clinical follow‐up. A particular focus was addressed to cases featuring a predominance of large cells, encompassing the whole spectrum of PCDLBCL according to both WHO and WHO/EORTC classifications. Thirty‐six cases were excluded because of a history of systemic lymphoma or limited follow‐up.

### Histological review

#### Immunohistochemistry

For all cases, histochemical and immunohistochemical staining was reviewed by a panel of six expert pathologists (M. P., E. B., C. T., S. A., M. G., and M. S.). Automated immunostainings were performed on FFPE slides through streptavidin‐biotin‐peroxidase‐conjugated (SABC) method after antigen retrieval procedures, when needed. Tested antibodies included CD20, CD79a, BCL2, CD10, BCL6, MUM1, MYC, HGAL, CD138, CD3, CD5, Mib1/Ki‐67, CD21, CD23, CD30, BCL1, and ALK/p80. BCL2, BCL6, and CD10 immunostainings were considered positive if >50% of the cells were stained. MUM1 positivity was assessed upon a cutoff value of 30%. MiB1/Ki67 expression was assigned to a low (<50%) or high proliferative (>50%) index. Histogenesis was defined according to Hans algorithm 12, and thus a “germinal center B‐cell” (GC) or a “non‐germinal center B‐cell” (non‐GC) profile was assigned. The so‐called double hit score (DHS) was assigned to DLBCL based on a cutoff value of 75% for BCL2 positivity and of 40% for MYC positivity 13.

Diagnoses were primarily based on the 2008 WHO classification criteria 1. When a disagreement occurred, final diagnosis was obtained by consensus. Lesional architecture was identified as nodular, nodular/diffuse, or diffuse; the presence of residual dendritic meshwork was noted. Cytologic features were defined primarily on nuclear morphology either as small‐to‐large centrocytes (cleaved cells) or as centroblasts and immunoblasts (nucleolated, noncleaved cells).

**Figure 1:**
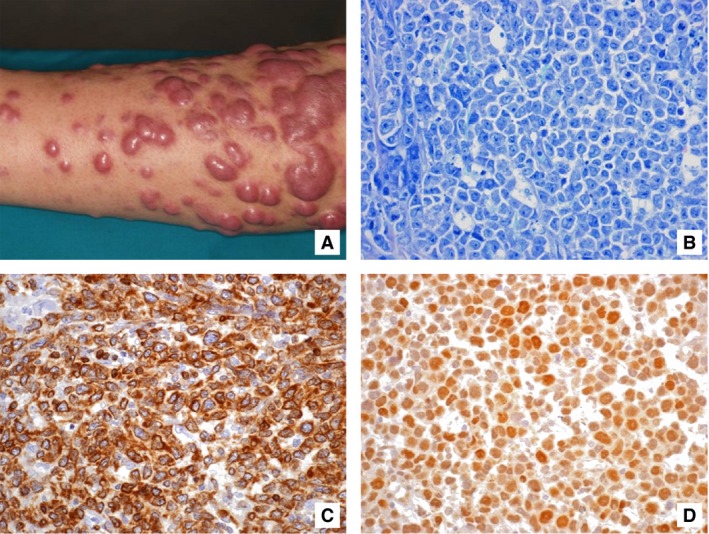
The typical picture of PCDLBCL‐LT is represented, as tumoral lesions arising on the lower limbs (A), composed of a proliferation of large, round cells with centroblastic and/or immunoblastic features (B; Giemsa stain, 400×) and frequent coexpression of BCL2 and MYC (C and D; SABC method, 400×). PCDLBCL‐LT, primary cutaneous diffuse large B‐cell lymphoma, leg type; BCL2, B‐cell lymphoma; SABC, streptavidin‐biotin‐peroxidase‐conjugated.

Cases with a predominance of small‐to‐large centrocytes and a minority of centroblasts/immunoblasts were classified as PCFCCL, independently from growth pattern (Fig. S1). Proliferations showing a diffuse pattern and mostly consisting of centroblasts/immunoblasts with only few small, centrocytoid lymphocytes were named PCDLBCL‐LT (Fig. 1).

**Figure 2:**
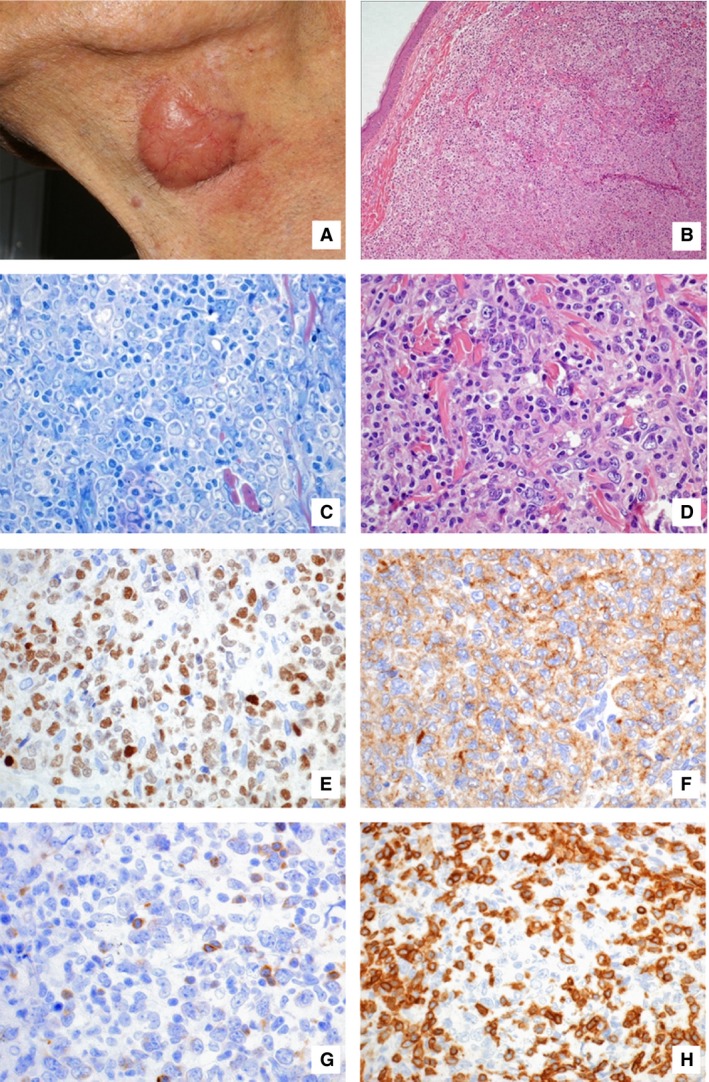
This case of PCDLBCL‐NOS arose as a tumoral lesion on the neck (A); histologic picture is consistent with a nodular to diffuse proliferation (B, hematoxylin–eosin, 100×) of predominantly large, centroblastic cells (C, Giemsa stain 400×) with a mixed inflammatory infiltrate (D, hematoxylin–eosin 400×). Picture (C) is representative of a PCDLBCL‐NOS‐non‐GC cases, which resulted MUM1‐positive (E, SABC method, 400×), whereas CD10 stain (F, SABC method, 400×) corresponds to the PCDLBCL‐NOS‐GC cases shown in picture (D). BCL2 is usually negative (G, SABC method, 400×), whereas the small, intermixed lymphocytes usually display a T‐cell, CD3+ phenotype (H, SABC method, 400×). PCDLBCL‐NOS‐non‐GC, primary cutaneous diffuse large B‐cell lymphoma, not otherwise specified non‐germinal center B‐cell; SABC, streptavidin‐biotin‐peroxidase‐conjugated.

Cases almost entirely composed of large cells (centroblasts), though with a mixed inflammatory background and/or a minority (<10%) of large centrocytoid cells, and with a predominantly diffuse pattern were observed (Fig. 2). These tumors lacked the typical features both of PCDLBCL‐LT and PCFCCL, and thus they were named PCDLBCL, not otherwise specified (PCDLBCL‐NOS).

### Molecular biology

Interphasic fluorescence in situ hybridization (FISH) analysis for *BCL*2 translocation was performed on routine paraffin sections (3–4 *μ*m) using an *IGH/BCL2* Dual Color, Dual Fusion Translocation Probe (Vysis Abbott, Des Plaines, IL, USA). This probe is a mixture of the *IGH* probe, labeled with SpectrumGreen and spanning ~1.5 Mb, thus containing sequences homologous to the entire *IGH* locus as well as sequences extending about 300 kb beyond the 3′‐end of the *IGH* locus, and the *BCL2* probe, labeled with SpectrumOrange and covering gene, covering an approximate 750‐kb region. The expected pattern in a normal nucleus hybridized is the two orange, two green; if harboring a t(14;18), the most common pattern is one orange signal, one green signal, and two orange/green (yellow) fusion signals, representing the two derivative chromosomes resulting from the reciprocal translocation. The evaluation was carried out using direct viewing on a standard fluorescence microscope, and the images were elaborated with Powergene Macprobe v.4.4 software (Applied Imaging, Newcastle‐upon‐Tyne, UK). In each case, more than 100 nuclei on paraffin‐embedded sections were examined; if more than 15% of nuclei displayed the translocation, we considered the case as positive.

Epstein‐Barr virus (EBV) status was tested by in situ hybridization (ISH) using a fluorescein isothiocyanate‐labeled peptic nucleic acid (PNA) probe, complementary to the EBV‐encoded RNAs (*EBER*s) (DakoCytomation, Glostrup, Denmark).

### Statistical analysis

Data were described as mean and standard deviation if continuous variable and counts and percent if categorical variable and compared between diagnostic groups with the one‐way analysis of variance and the Fisher exact test, respectively. Survival and event‐free survival were described with Kaplan–Meier method. Predictors were identified with the log‐rank test, and the Cox model was used to compute the corresponding hazard ratios and their 95% confidence intervals (HR, 95% CI). The analysis was performed on the entire case series and on predefined meaningful subgroups. The median follow‐up (25th–75th percentiles) was computed according to the inverse Kaplan–Meier method.

Stata13 (StataCorp, College Station, TX) was used for computation. A two‐sided *P*‐value was considered statistically significant. For post hoc comparisons, the Bonferroni correction was applied.

## Results

### Histological classification

According to the panel approach, 96/161 cases (59%) were classified as PCFCCL, 40/161 (25%) as PCDLBCL‐NOS, and 25/161 (16%) as PCDLBCL‐LT.

Briefly, in PCFCCL (Fig. S1), the infiltrate mainly consisted of small‐ to medium‐sized centrocytes, with a variable amount of centroblasts, whereas large cells (both centrocytes and centroblasts) were predominant in 20/96 (21%) cases. A spindle cell morphology was observed in 11 cases. Growth pattern was nodular in 33/96 (34%) cases, nodular and diffuse in 39/96 (41%), and purely diffuse in 24/96 (25%), whereas remnants of follicular dendritic meshwork were usually observed. A reactive lymphocytic and histiocytic background was always present, at times so abundant to obscure the lymphoma B cells.

In PCDLBCL‐NOS (Fig. 2), the infiltrate showed a purely diffuse growth pattern in 25/40 (63%) cases, while limited gross nodular areas were observed in 15/25 (37%) cases; in 11/40 (27%) cases, a residual dendritic meshwork was noted, though very focal and with features of disruption. Tumor cells were chiefly centroblasts and were usually intermingled with a variable reactive cellular background, mostly composed of small reactive CD3+ lymphocytes.

PCDLBCL‐LT (Fig. 1) was composed exclusively of large round nucleolated cells, with predominance of immunoblasts, growing in a diffuse pattern with common effacement of adnexa, focal necrosis with sparse nuclear debris, and a very scanty, if present, inflammatory background nor stromal reaction; no dendritic meshwork was detected.

Although within a wide range, median proliferative index was generally low in PCFCCL (30%, range 10–90%) and high in PCDLBCL‐LT (70%, range 50–90%), whereas an intermediate value was documented in PCDLBCL‐NOS (50%, range 10–90%).

All PCFCCLs were positive for either CD10 or Bcl6 and negative for MUM1, whereas PCDLBCL subtypes were split in the two histogenetic groups. For PCDLBCL‐NOS, 26/40 (65%) cases were recorded as GC and 14/40 (35%) as non‐GC; among PCDLBCL‐LT, 5/25 (20%) cases fell into GC and 20/25 (80%) into non‐GC subgroup. MYC positivity was documented in 10 of 21 (48%) tested cases of PCDLBCL‐NOS and in 11/13 (85%) PCDLBCL‐LT, whereas it turned out to be negative in PCFCCL. As to DHS, cases were stratified into a two‐tiered system (0–1 vs. 2): within PCDLBCL‐NOS, 16/24 cases scored DHS = 0–1 and 8/24 DHS = 2; among PCDLBCL‐LT, 18 cases 5/18 cases scored DHS = 0–1 and 13/18 DHS = 2. Comprehensive histopathologic and phenotypic features are detailed in Table 1.

**Table 1:** Histologic features

Histopathologic features	PCFCCL	PCDLBCL‐NOS	PCDLBCL‐LT	*P*
Cytology	Prevalence of small to large, cleaved cells (centrocytes)	Prevalence of round, nucleolated cells (centroblasts, rarely immunoblasts)	Almost exclusively round, nucleolated cells (centroblasts and immunoblasts)	—
Reactive T cells	Present	Present	Very scanty	—
Growth pattern (%)	Nodular to diffuse	Typically diffuse	Diffuse	NA
Nodular	33/96 (34)	0/40 (0)	0/25 (0)	
Nodular/diffuse	39/96 (41)	15/40 (38)	0/25 (0)	
Diffuse	24/96 (25)	25/40 (62)	25/25 (100)	
Dendritic meshwork, present (%)	80/96 (83)	11/40 (27)^a^	1/25 (4)^a^	<0.001
Infiltrate extension (%)				<0.001
Dermic	44/96 (46)	22/40 (55)	0/25 (0)	
Dermic/hypodermic	52/96 (54)	18/40 (45)	25/25 (100)	
Skin ulceration (%)	0/96 (0)	4/40 (10)	4/25 (16)	NA
Adnexal effacement, present (%)	3/96 (3)	6/40 (15)	10/25 (40)	NA
Necrosis	0/96 (0)	2/40 (5)	4/25 (16)	NA
Nuclear debris	0/96 (0)	4/40 (10)	15/25 (60)	NA
Starry sky appearance	0/96 (0)	0/40 (0)	11/25 (44)	NA
BCL2, +/total (%)	29/96 (30)	16/40 (40)	19/25 (76)	<0.001
CD10, +/total (%)	57/96 (59)	11/40 (27)	0/25 (0)	<0.001
BCL6, +/total (%)	84/96 (87)	33/40 (82)	14/25 (56)	0.001
MUM1, +/total (%)	0/96 (0)	14/40 (40)	20/25 (80)	0.004
HGAL, +/total (%)	50/54 (93)	9/40 (22)	1/25 (4)	<0.001
MYC, +/total (%)	0/40 (0)	10/21 (48)	11/13 (85)	<0.001
Ki67 median % (range)	30 (10–90)	50 (10–90)	70 (50–90)	—
Histogenetic profile, GC/total (%)	96/96 (100)	26/40 (65)	5/25 (20)	<0.001
DHS (%)				All: <0.001
0–1	NA	16/24	5/18	NOS vs. LT: 0.28
2	NA	8/24	13/18	
*BCL2* translocation +/total (%)	15/75 (20)	3/27 (11)	1/20 (5)	0.234
BCL2 status (p)	(<0.001)	(0.273)	(1)	—
FISH+/IHC+ (%)	11/23 (48)	3/17 (17)	1/17 (6)	
FISH+/IHC− (%)	4/52 (8)	0/10 (0)	0/3 (0)	
EBV, +/total (%)	NA	0/15 (0)	0/20 (0)	NA

PCFCCL, primary cutaneous follicular center cell lymphoma; PCDLBCL‐NOS, primary cutaneous diffuse large B‐cell lymphoma, not otherwise specified; PCDLBCL‐LT, primary cutaneous diffuse large B‐cell lymphoma, leg type; GC, germinal center (Hans algorithm); DHS, double‐hit score; NA, not assessed (group too small for statistical analysis); FISH, fluorescence in situ hybridization; IHC, immunohistochemistry.

a Only very focal and disrupted, if present.

### Molecular biology

FISH analysis for *BCL2* translocation was performed in 122/161 (76%) cases (Table 1) and detected in 15/75 (20%) PCFCCL, in 3/27 (11%) PCDLBCL‐NOS, and in 1/20 (5%) PCDLBCL‐LT. EBV was tested in 30 PCDLBCL and resulted uniformly negative (Fig. S2).

### Clinical presentation, therapy, and follow‐up

Clinical features, therapy, and follow‐up are summarized according to the panel diagnosis and detailed in Table 2. Among the three groups, a slight male‐to‐female prevalence was noticed; for PCDLBCL‐LT, a tendency toward an older age of onset was highlighted. The number of lesions (single vs. multiple) was balanced among the subgroups, whereas PCFCCL and PCDLBCL‐NOS showed a predilection for trunk and head and neck location, in contrast to PCDLBCL‐LT which involved preferentially the lower limbs.

**Table 2:** Clinical features

Clinical presentation	PCFCCL	PCDLBCL‐NOS	PCDLBCL‐LT (%)	*P*
Male/female (ratio)	53/43 (1.23)	27/13 (2.08)	17/8 (2.12)	0.432
Mean age (range)	54 (27–86)	63 (26–90)	76 (54–92)	<0.001
Number of lesions (%)				
Single lesion	67/96 (70)	27/40 (68)	18/25 (72)	0.889
Multiple lesions	29/96 (30)	13/40 (32)	6/25 (24)	
Diffuse	0/96 (0)	0/40 (0)	1/25 (4)	
Site involved (%)				
Head and neck	38/96 (40)	7/40 (17)	0/25 (0)	<0.001
Trunk	47/96 (49)	20/40 (50)	3/25 (12)	0.002
Upper limbs	8/96 (8)	8/40 (20)	1/25 (4)	NA
Lower limbs	7/96 (7)	9/40 (22)	21/25 (84)	<0.001
Type of lesion (%)				
Nodule/tumor	64/96 (67)	27/40 (67)	18/25 (72)	0.878
Plaque	17/96 (18)	10/40 (25)	5/25 (20)	0.625
Patch	4/96 (4)	1/40 (3)	2/25 (8)	NA
Papule	5/96 (5)	0/40 (0)	0/25 (0)	NA
Variable	6/96 (6)	2/40 (5)	0/25 (0)	NA
Therapy and follow‐up				
First‐line therapy				
Surgical only	20/96 (21)	2/40 (5)	0/25 (0)	0.004
Radiotherapy	47/96 (49)	15/40 (37)	9/25 (36)	<0.001
Chemotherapy (±radio)	26/96 (27)	22/40 (55)	15/25 (60)	0.006
Wait and see	3/96 (3)	1/40 (3)	1/25 (4)	NA
Response to therapy (%)				
CR	81/96 (84)	32/40 (80)	13/25 (52)	0.002
PR	15/96 (16)	8/40 (20)	12/25 (48)	
Relapse, /CR (%)	35/81 (43)	13/32 (41)	11/13 (85)	0.015
Extracutaneous relapse, /CR (%)	5/81 (6)	2/32 (6)	1/25 (4)	
Median time to relapse, months (range)	24 (6–156)	26 (5–159)	11 (5–28)	0.156
Follow‐up				
ADF	76/96 (79)	25/40 (62)	4/25 (16)	<0.001
AWD	15/96 (16)	10/40 (25)	8/25 (32)	0.140
DOD	2/96 (2)	4/40 (10)	11/25 (44)	NA
DUC	3/96 (3)	1/40 (3)	2/25 (8)	NA
Median follow‐up, months (range)	47 (12–237)	53 (8–210)	19 (6–126)	0.007

PCFCCL, primary cutaneous follicular center cell lymphoma; PCDLBCL‐NOS, primary cutaneous diffuse large B‐cell lymphoma, not otherwise specified; PCDLBCL‐LT, primary cutaneous diffuse large B‐cell lymphoma, leg type; CR, complete response; PR, partial response; ADF, alive disease‐free; AWD, alive with disease; DOD, died of disease; DUC, died of unrelated cause; NA, not assessed (group too small for statistical analysis).

### Overall survival

**Figure 3:**
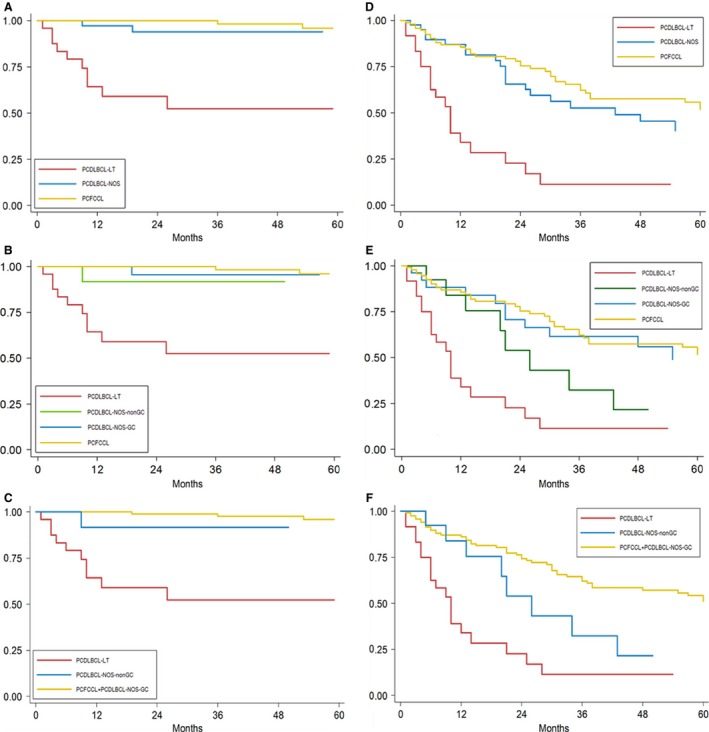
OS curves: analysis is performed comparing the three morphologic diagnosis, according to (A), the four groups obtained when splitting PCDLBCL‐NOS according to histogenesis (B) and the three categories identified upon aggregation of PCFCCL and PCDLBCL‐NOS‐GC in a “germinal center” group (C); in the same way, EFS curves are reported (D–F). OS, overall survival; PCDLBCL‐NOS‐GC, primary cutaneous diffuse large B‐cell lymphoma, not otherwise specified germinal center B‐cell; PCFCCL, primary cutaneous follicular center cell lymphoma; EFS, event‐free survival.

On the whole series, the median follow‐up was 48 months (25th–75th, 21–98). Median overall survival (OS) was not reached for any subgroup. According to the panel diagnosis (Fig. 3, Table 3), OS was significantly different between the three subgroups (*P* < 0.001). Post hoc comparisons scored a significantly different OS for PCFCCL versus PCDLBCL‐LT (HR = 0.03, *P* < 0.001) and for PCDLBCL‐NOS versus PCDLBCL‐LT (HR = 0.13, *P* = 0.001); however, the comparison of PCFCCL versus PCDLBCL‐NOS did not reach statistical significance (HR = 0.21, *P* = 0.073). When splitting PCDLBCL‐NOS in two histogenetic subgroups, post hoc comparisons showed a difference in OS for PCDLBCL‐NOS‐GC versus PCDLBCL‐NOS‐non‐GC, although not significant (HR = 0.15, *P* = 0.102). The difference in OS of PCDLBCL‐NOS‐GC was significant versus PCDLBCL‐LT (HR = 0.05, *P* = 0.003) but not versus PCFCCL (HR = 0.58, *P* = 0.695). For PCDLBCL‐NOS‐non‐GC, the comparison did not reach statistical significance versus PCDLBCL‐LT (HR = 0.30, *P* = 0.070) but versus PCFCCL (HR = 0.09, *P* = 0.008).

**Table 3:** OS and EFS according to the diagnosis and paired comparisons

	2‐year OS %	5‐year OS %	*P*	Paired comparisons	HR	*P*
Analysis of OS by panel diagnosis						
PCFCCL	100	98.25	<0.001	PCFCCL vs. PCDLBCL‐LT	0.03 (0.01–0.12)	<0.001
PCDLBCL‐NOS	93.98	93.98		PCDLBCL‐NOS vs. PCDLBCL‐LT	0.13 (0.04–0.41)	0.001
PCDLBCL‐LT	58.96	52.41		PCFCCL vs. PCDLBCL‐NOS	0.21 (0.04–1.16)	0.073
Analysis of OS by panel diagnosis + histogenesis						
PCFCCL	100	95.96	<0.001	PCFCCL vs. PCDLBCL‐LT	0.03 (0.01–0.12)	<0.001
PCDLBCL‐NOS‐GC	95.45	95.45		PCFCCL vs. PCDLBCL‐NOS‐GC	0.58 (0.05–6.42)	0.695
PCDLBCL‐NOS‐non‐GC	91.67	91.67		PCFCCL vs. PCDLBCL‐NOS‐non‐GC	0.09 (0.01–0.53)	0.008
PCFLBCL‐LT	58.96	52.41		PCDLBCL‐NOS‐GC vs. PCDLBCL‐LT	0.05 (0.01–0.36)	0.003
				PCDLBCL‐NOS‐non‐GC vs. PCDLBCL‐LT	0.30 (0.08–1.11)	0.070
				PCDLBCL‐NOS‐GC vs. PCDLBCL‐NOS‐non‐GC	0.15 (0.02–1.45)	0.102
Analysis of OS by combined groups						
PCFCCL+PCDLBCL‐NOS‐GC	98.95	95.90	<0.001	PCFCCL+PCDLBCL‐NOS‐GC vs. PCDLBCL‐LT	0.03 (0.01–0.11)	<0.001
PCDLBCL‐NOS‐non‐GC	91.96	91.96		PCDLBCL‐NOS‐non‐GC vs. PCFLBCL‐LT	0.30 (0.08–1.11)	0.070
PCDLBCL‐LT	58.96	52.41		PCFCCL+PCDLBCL‐NOS‐GC vs. PCDLBCL‐NOS‐non‐GC	0.10 (0.02–0.51)	0.005
Analysis of EFS by panel diagnosis						
PCFCCL	75.29	51.67	<0.001	PCFCCL vs. PCDLBCL‐LT	0.21 (0.12–0.37)	<0.001
PCDLBCL‐NOS	65.69	40.30		PCDLBCL‐NOS vs. PCDLBCL‐LT	0.24 (0.13–0.47)	<0.001
PCDLBCL‐LT	22.69	11.34		PCFCCL vs. PCDLBCL‐NOS	0.86 (0.50–1.47)	0.582
Analysis of EFS by panel diagnosis + histogenesis						
PCFCCL	75.29	51.67	<0.001	PCFCCL vs. PCDLBCL‐LT	0.21 (0.12–0.37)	<0.001
PCDLBCL‐NOS‐GC	70.69	48.95		PCFCCL vs. PCDLBCL‐NOS‐GC	1.08 (0.56–2.07)	0.817
PCDLBCL‐NOS‐non‐GC	53.95	21.58		PCFCCL vs. PCDLBCL‐NOS‐non‐GC	0.54 (0.25–1.16)	0.113
PCFLBCL‐LT	22.69	11.34		PCDLBCL‐NOS‐GC vs. PCDLBCL‐LT	0.19 (0.09–0.41)	<0.001
				PCDLBCL‐NOS‐non‐GC vs. PCDLBCL‐LT	0.38 (0.17–0.88)	0.024
				PCDLBCL‐NOS‐GC vs. PCDLBCL‐NOS‐non‐GC	0.50 (0.20–1.23)	0.135
Analysis of EFS by combined groups						
PCFCCL+PCDLBCL‐NOS‐GC	74.27	51.13	<0.001	PCFCCL+PCDLBCL‐NOS‐GC vs. PCDLBCL‐LT	0.20 (0.12–0.35)	<0.001
PCDLBCL‐NOS‐non‐GC	53.95	21.58		PCDLBCL‐NOS‐non‐GC vs. PCFLBCL‐LT	0.38 (0.17–0.88)	0.024
PCDLBCL‐LT	22.69	11.34		PCFCCL+PCDLBCL‐NOS‐GC vs. PCDLBCL‐NOS‐non‐GC	0.53 (0.25–1.12)	0.098

OS, overall survival; EFS, event‐free survival; HR, hazard ratio; PCFCCL, primary cutaneous follicular center cell lymphoma; PCDLBCL‐NOS, primary cutaneous diffuse large B‐cell lymphoma, not otherwise specified; PCDLBCL‐LT, primary cutaneous diffuse large B‐cell lymphoma, leg type; PCDLBCL‐NOS‐GC, pCDLBCL‐NOS germinal center B‐cell; PCDLBCL‐NOS‐non‐GC, PCDLBCL‐NOS non‐germinal center B‐cell.

The combination of PCFCCL and PCDLBCL‐NOS‐GC into a “germinal center” group was tested: this approach identified for PCFCCL+PCDLBCL‐NOS‐GC a significantly different OS as compared to the “high‐grade” subgroup, identified as PCDLBCL‐NOS‐non‐GC+PCDLBCL‐LT (HR 0.05, *P* < 0.001). Interestingly, statistic significance was retained also toward, respectively, PCDLBCL‐NOS‐non‐GC (HR = 0.10, *P* = 0.005) and PCDLBCL‐LT (HR = 0.03, *P* = 0.001).

### Event‐free survival

Event‐free survival (EFS) (Fig. 3, Table 3) was significantly different between the three panel diagnosis (*P* < 0.001). Post hoc analysis showed a significantly different EFS only for PCFCCL versus PCDLBCL‐LT (HR = 0.21, *P* < 0.001) and for PCDLBCL‐NOS versus PCDLBCL‐LT (HR = 0.24, *P* < 0.001).

As to histogenetic subsets, paired comparison resulted in a significantly different EFS only for PCDLBCL‐NOS‐GC versus PCDLBCL‐LT (HR = 0.19, *P* < 0.001) and for PCDLBCL‐NOS‐non‐GC versus PCDLBCL‐LT (HR = 0.38, *P* = 0.024).

Finally, PCFCCL+PCDLBCL‐NOS‐GC group had a higher EFS when compared to the “high‐grade” group (HR = 0.31, *P* < 0.001) and to PCDLBCL‐LT (HR = 0.20, *P* < 0.001), while EFS versus PCDLBCL‐NOS‐non‐GC was still lower but not significant (HR = 0.53, *P* = 0.098).

### Survival according to single factors

Univariable analysis is detailed in Table 4; a further testing was conducted on the group of PCDLBCL (PCDLBCL‐NOS+PCDLBCL‐LT).

**Table 4:** Univariable analysis

Parameter	All			PCFCCL			PCDLBCL‐NOS			PCDLBCL‐LT			PCDLBCL‐NOS+PCDLBCL‐LT		
	5 years %	HR (CI)	*P*	5 years %	HR (CI)	*P*	5 years %	HR (CI)	*P*	5 years %	HR (CI)	*P*	5 years %	HR (CI)	*P*
Overall survival															
Sex															
F	90.08	—	0.381												
M	88.14	1.60 (0.56–4.54)													
Age															
<70 years	95.98	—	0.003												
>70 years	80.94	9.51 (2.17–41.62)													
Histogenesis															
GC	94.12	—	<0.001												
Non‐GC	69.59	0.07 (0.03–0.21)													
Bcl2 IHC															
−	94.58	—	0.001	96.88	—	0.348	95.00	—	0.242	66.67	—	0.628	88.67	—	0.043
+	77.74	5.74 (1.99–16.57)		90.91	3.77 (0.24–60.37)		92.31	3.43 (0.44–27.00)		53.03	0.67 (0.14–3.31)		69.73	3.40 (1.04–11.09)	
DHS															
0–1	94.59	—	<0.001	NA	NA	NA	92.86	—	0.919	80.00	—	0.322	89.64	—	0.053
2	53.59	13.15 (4.37–39.63)					83.33	1.12 (0.12–10.98)		36.46	2.89 (0.35–23.50)		53.59	3.43 (0.98–11.95)	
Bcl2 translocation															
Absent	91.66	—	0.502	100	—	0.020	94.12	NA	NA	NA	NA	NA	80.52	—	0.594
Present	75.56	1.71 (0.36–8.13)		80.00	19.24 (3.21–115.44)		100						75.00	1.77 (0.22–14.49)	
Number of lesions															
Single	88.67	—	0.895	93.48	—	1.000	95.24	—	0.512	59.89	—	0.557	82.23	—	0.494
Multiple	89.21	1.07 (0.40–2.90)		100	0.00 (0.00)		90.91	1.93 (0.27–13.83)		42.86	1.47 (0.41–5.27)		72.69	1.44 (0.51–4.04)	
Leg															
No	94.48	—	<0.001	97.56	—	0.037	92.57	—	0.554	60.00	—	0.620	87.23	—	0.013
Yes	68.34	9.64 (3.52–26.40)		66.67	19.21 (1.20–307.56)		100	2.01 (0.20–20.07)		51.81	1.49 (0.31–7.11)		67.58	3.97 (1.33–11.81)	
Anatomic site															
Lower limbs	68.34	—	—	66.67	—	—	100	—	—	51.81	—	—	67.58	—	—
Upper limbs	92.86	0.19 (0.02–1.49)	0.114	100	0.00 (0.00)	1.000	100	0.00 (0.00)	1.000	0.00	4.63 (0.51–41.68)	0.172	87.50	0.27 (0.03–2.11)	0.211
Trunk	95.13	0.11 (0.03–0.38)	0.001	95.00	0.11 (0.01–1.72)	0.115	93.75	0.58 (0.05–6.61)	0.658	100	0.00 (0.00)	1.000	94.74	0.17 (0.04–0.80)	0.025
Head/neck	97.30	0.04 (0.01–0.31)	0.002	100	0.00 (0.00)	1.000	85.71	0.70 (0.04–11.45)	0.803	NA	NA	NA	85.71	0.23 (0.03–1.82)	0.163
Event‐free survival															
Sex															
F	53.56	—	0.044												
M	35.39	1.63 (1.01–2.62)													
Age															
<70 years	47.64	—	0.167												
>70 years	36.69	1.37 (0.88–2.13)													
Histogenesis															
GC	49.61	—	<0.001												
Non‐GC	12.65	0.32 (0.19–0.53)													
Bcl2 IHC															
−	45.60	—	0.177	51.33	—	0.836	36.32	—	0.531	0.00	—	0.083	31.68	—	0.602
+	35.49	1.37 (0.87–2.18)		47.37	1.08 (0.51–2.29)		49.23	0.73 (0.28–1.93)		12.96	0.32 (0.09–1.16)		28.34	1.19 (0.62–2.26)	
DHS															
0–1	45.07	—	0.011	NA	NA	NA	28.57	—	0.741	30.00	—	0.454	22.71	—	0.334
2	28.42	2.20 (1.19–4.06)					57.14	0.80 (0.22–2.97)		11.00	0.63 (0.19–2.10)		28.42	1.46 (0.68–3.18)	
Bcl2 translocation															
Absent	42.57	—	0.324	50.39	—	0.210	47.44	—	0.677	13.40	—	0.037	31.55	—	0.773
Present	26.23	1.41 (0.71–2.79)		29.09	1.72 (0.74–4.02)		33.33	1.39 (0.30–6.49)		0.00	19.00 (1.19–303.76)		25.00	1.19 (0.36–3.98)	
Number of lesions															
Single	43.16	—	0.756	53.06	—	0.637	41.49	—	0.830	8.99	—	0.487	28.98	—	0.990
Multiple	39.94	1.08 (0.67–1.72)		48.64	1.17 (0.61–2.21)		27.20	0.90 (0.34–2.37)		14.29	1.42 (0.53–3.76)		20.20	1.00 (0.50–1.98)	
Leg															
No	49.40	—	<0.001	22.22	—	0.113	24.31	—	0.432	20.00	—	0.465	40.05	—	0.005
Yes	14.83	3.18 (1.91–5.28)		53.61	2.32 (0.82–6.59)		43.87	1.57 (0.51–4.83)		8.04	1.53 (0.49–4.81)		13.13	2.63 (1.34.5.15)	
Anatomic site															
Lower limbs	14.82	—	—	22.22	—	—	24.31	—	—	8.04	—	—	13.13	—	—
Upper limbs	24.19	0.64 (0.39–1.37)	0.248	0.00	1.20 (0.29–4.86)	0.802	35.71	1.14 (0.30–4.42)	0.846	0.00	2.26 (0.28–18.15)	0.444	31.25	0.67 (0.26–1.76)	0.418
Trunk	51.97	0.33 (0.18–0.57)	<0.001	53.58	0.57 (0.19–1.70)	0.315	53.72	0.50 (0.14–1.74)	0.276	33.33	0.40 (0.08–1.91)	0.252	50.18	0.29 (0.13–0.66)	0.003
Head/neck	55.97	0.22 (0.11–0.41)	<0.001	61.24	0.27 (0.09–0.84)	0.023	26.79	0.61 (0.15–2.49)	0.493	NA	NA	NA	26.79	0.31 (0.10–0.94)	0.039

HR, hazard ratio; PCFCCL, primary cutaneous follicular center cell lymphoma; PCDLBCL‐NOS, primary cutaneous diffuse large B‐cell lymphoma, not otherwise specified; PCDLBCL‐LT, primary cutaneous diffuse large B‐cell lymphoma, leg type; GC germinal center B‐cell; non‐GC, non‐germinal center B‐cell; IHC, immunohistochemistry; DHS, double‐hit score; NA, not assessed (group too small for statistical analysis).

As to immunophenotypic features, on the complete series, a GC histogenetic profile distinguishes a whole group both with a better OS (HR = 0.07, *P* < 0.001) and EFS (HR = 0.32, *P* < 0.001); this observation was true also excluding PCDLBCL‐LT cases from the analysis. BCL2 positivity negatively impacted OS but not EFS on the whole series. According to the panel diagnosis, there was a trend toward ad increase in OS, though above the threshold of significance while an inverse tendency, though still not significant, was observed for EFS. OS was significantly impacted when considering only cases with a large cell histology (HR = 3.43, *P* = 0.043). DHS proved to impact OS (*P* < 0.001) and EFS (*P* = 0.011) on the whole series and to be helpful in identifying a subset of cases with a lower survival in the large cell subgroup (HR = 3.43, *P* = 0.05).

Although based on few events, the presence of *BCL2* translocation proved to impact significantly OS in PCFCCL, whereas for EFS, it was significant only for PCDLBCL‐LT.

Age at diagnosis >70 years correlated with a significantly lower OS (HR = 9.51, *P* = 0.003). Male sex resulted to be a factor of risk, although statistically significant only for EFS.

Lesional pattern (single vs. multiple lesions) did not show any significant impact. Localization on the lower limbs correlated with worse OS and EFS on the whole series, whereas according to the panel diagnosis, it was significant only for PCFCCL, however based on a single event. A significance was observed also in the large cell subgroup, with a lower OS and HR = 3.97 (*P* = 0.013) for leg site, whereas the highest OS was related to localization on the trunk.

## Discussion

The controversies in PCBCL classification primarily reflect the rarity and clinical heterogeneity of the disease. From the histopathologist's standpoint, the major challenge is the proper classification of PCBCL displaying a diffuse pattern and a predominant large cell histology.

We defined PCDLBCL‐NOS as a subset of cases exhibiting diffuse large B‐cell histology, not fitting into PCFCCL diffuse type subgroup nor in PCDLBCL‐LT both in cytology and in phenotype. PCDLBCL‐NOS predominantly consisted of centroblasts, often intermingled with a brisk infiltrate of small lymphocytes, which are usually inconspicuous in PCDLBCL‐LT. However, PCDLBCL‐NOS differed from PCFCCL because large centrocytoid cells represented only a limited fraction (<10%) of the infiltrate, whereas no dendritic meshwork was detectable other than minimal remnants (in a minority of cases). Phenotypically, they variably expressed MYC and BCL2 that were intensely coexpressed in PCDLBCL‐LT. PCDLBCL‐NOS partially overlapped with the subset of PCDLBCL‐other as described in the 2005 WHO/EORTC classification 7, 8 and by Kodama et al. 14, where they are reported to have histologic features in between PCFCCL and PCDLBCL‐LT, showing predominance of round cells and variable BCL2 expression.

We aimed to clarify whether PCDLBCL‐NOS represents a distinct clinicopathologic subset or simply a morphophenotypic variant of PCFCCL and/or PCDLBCL‐LT by analyzing their outcome. Comparison of PCDLBCL‐NOS as a whole with PCFCCL resulted in a difference in OS, though below the threshold of significance. Separation of PCDLBCL‐NOS upon histogenetic profile documented a worse prognosis for the non‐GC subgroup, whereas cases with a GC profile were more similar to PCFCCL. Since PCDLBCL‐NOS with a GC profile cannot be distinguished from the more aggressive PCDLBCL‐NOS with a non‐GC profile on the sole morphological ground, we think that a more accurate prognostic stratification of this category should rely on the immunophenotypic and/or molecular characterization. As well, cases of PCDLBCL‐NOS with a non‐GC profile would be classified by some pathologists as PCFCCL 15 with high content of blast cells, but they are different in terms of both clinical course and outcome (shorter survival) as compared to PCFCCL. Although the small number of cases of PCDLBCL‐NOS with a non‐GC phenotype did not allow us to reach a statistical significance when comparing their outcome to PCDLBCL‐LT, a trend toward a less aggressive course was observed. Notably, PCDLBCL‐NOS‐non‐GC clearly differs from PCDLBCL‐LT in terms of presentation site, cytologic features (centroblasts with an intermixed reactive infiltrate), and phenotype (rare MYC/BCL2 coexpression).

Since the description of PCDLBCL‐LT by Vermeer et al. 16, the concept of PCDLBCL has been tightly connected to a specific anatomic location on the lower limbs, as well as the “leg” involvement denoted a poor prognostic indicator. Further series reported an analogous behavior for PCDLBCL‐LT arising at different sites 3. Our study highlights that the prognostic role of the leg location is retained on the whole series but not in PCDLBCL‐LT alone, which in turn arises more frequently in the lower extremities. This finding suggests that histopathology and other biologic factors rather than “leg” location only might be predictive of a potential aggressive behavior.

First‐line treatment was mainly radiotherapy in PCFCCL (49%) and chemotherapy (±local radiotherapy) in both PCDLBCL‐LT (60%) and PCDLBCL‐NOS (55%). However, follow‐up data of PCDLBCL‐NOS were more similar to PCFCCL and complete response and relapse rate and number of patients alive free of disease consistently differed from PCDLBCL‐LT (Table 2). With the limitations of a retrospective data collection, these observations suggest the opportunity of a radiotherapy‐privileged first‐line treatment for PCDLBCL‐NOS, particularly in cases with a GC profile.

PCFCCL and PCDLBCL‐LT harbor different molecular profiles 4, 5, 6, 17, 18; however, only limited data are available on the above‐mentioned subset with features in between PCFCCL and PCDLBCL‐LT 19. We applied Hans algorithm, as a surrogate of gene expression profiling (GEP) to define the histogenesis, and DHS to test the prognostic impact of two immunohistochemical algorithms validated in the diagnostic workup of systemic DLBCL 12, 13. We are well aware that immunohistochemical algorithms remain an imperfect substitution of GEP, partly due to their inherent oversimplification; nonetheless they provide a practical way of designating subtype and may be sufficient for the purpose of achieving population enrichment on clinical trials, although being less reliable for individual patient management 20. However, our results seem to enhance the concept of cell‐of‐origin and its prognostic relevance also in the setting of PCBCL, since we distinguished PCDLBCL‐NOS with a non‐GC phenotype as having an intermediate behavior between classic PCFCCL and PCDLBCL‐LT. As to DHS, though basing on a limited number of cases, BCL2/MYC coexpression proved helpful to identify cases with a more aggressive course among the whole group of PCDLBCLs, in a way independent from the histology. The latter observation was confirmed also for the sole BCL2 positivity. However, the definition of the genetic landscape of PCDLBCL‐NOS in comparison with PCDLBCL‐LT and PCFCCL could be a matter of future interest.

Currently no widely accepted prognostic indicators exist for PCFCCL. Similarly to previous reports, our series of PCFCCL showed an excellent prognosis with a 5‐year disease‐specific survival over 95% and only two patients dead of progression to systemic lymphoma. In our series, leg presentation and presence of t(14;18)(q32;q21) adversely affected prognosis. Whereas the former has been already associated with a more aggressive course 3, the prognostic role of t(14:18) is still debated.

t(14;18)(q32;q21) involves *BCL2* and *IGH* and represents the cytogenetic hallmark of nodal follicular lymphoma, whereas its detection in PCFCCL requires to exclude a secondary localization 21. *BCL2* translocation has been variably detected in PCFCCL both in studies using polymerase chain reaction (PCR)‐based methods (0–34%) and FISH analysis (0–41%) (Table 5) 21, 22, 23, 24, 25, 26, 27, 28, 29, 30, 31, 32, 33. Possible explanations for this wide range include geographic distribution, the limited number and heterogeneity of at least some of the reported series, and variation in the diagnostic criteria in different studies, probably including cases of skin involvement in the course of systemic follicular lymphoma. The clinical relevance of *BCL2* rearrangement in PCFCCL is controversial. Abdul‐Wahab et al. 32 reported that chromosomal anomalies, including t(14;18), do not portend a poor prognosis, as *BCL2*‐translocated patients do not differ in terms of clinical outcome and invariably respond to radiotherapy. On the contrary, Pharm Ledard et al. 33 reported that *BCL2* rearrangement correlates to a higher risk of extracutaneous spread.

**Table 5:** Comparison of BCL2 evaluation in PCFCCL among series

Evaluation of BCL2	IHC	PCR	FISH
Cerroni et al., 2000 22	0/15	0/15	NA
Franco et al., 2001 21	11/18 (61%)	0/18	NA
Bergman et al., 2001 23	4/19 (21%)	2/15 (13%)	NA
Aguilera et al., 2001 24	11/18 (61%)	3/17 (18%)	NA
Child et al., 2001 25	0/25	0/25	NA
Lawnicki et al., 2002 26	8/20 (40%)	4/20 (20%)	NA
Goodlad et al. 2002 27	3/16 (81%)	0/16	NA
Mirza et al., 2002 28	13/32 (41%)	11/32 (34%)	NA
Vergier et al., 2004 29	17/30 (57%)	9/30 (30%)	0/17
Kim et al., 2005 30	17/30 (57%)	NA	4/13 (31%)
Streubel et al., 2006 31	10/27 (37%)	0/17	11/27 (41%)
Abdul‐Wahab et al., 2014 [32	6/57 (11%)	NA	4/49 (8%)
Pharm Ledard et al., 2015 33	25/47 (53%)	NA	4/47 (8.5%)
Present series	29/96 (30%)	NA	15/75 (20%)

BCL2, B‐cell lymphoma; PCFCCL, primary cutaneous follicular center cell lymphoma; IHC, immunohistochemistry; PCR, polymerase chain reaction; FISH, fluorescence in situ hybridization; NA, not assessed.

To the best of our knowledge, the present series is the largest ever tested for *BCL2* rearrangement, encompassing the entire histologic spectrum of PCFCCL according to the WHO classification. FISH was preferred, due to its higher sensitivity for detection of *IGH/BCL2* rearrangement than PCR 31. We documented t(14;18)(q32;q21) in 15/75 (18%) patients, of which seven patients experienced cutaneous relapses and one patient died after systemic progression. Discordance between the presence of *BCL2* translocation and protein expression is a well‐reported occurrence in a limited fraction of systemic FL, which may lie with mutational events at *BCL2* locus 34. We tested our cases using BCL2 clone 124, and a significant correlation was found between protein expression and t(14;18), since it occurred in 48% BCL2‐positive cases but only in 8% BCL2‐negative cases (*P* < 0.001). While the presence of t(14;18) was associated with decreased OS, BCL2 expression did not seem to affect prognosis: as a consequence, FISH analysis could be included in the PCFCCL work‐up, to identify patients requiring closer monitoring.

Our findings indicate that PCLBCL includes different subsets, among which the so‐called leg type probably represents an aggressive clinical variant; a further group may exist, exhibiting clinicopathologic features intermediate between PCFCCL and PCDLBCL‐LT. Careful combination of morphological and immunophenotypic criteria with adequate clinical information is crucial to identify such cases.

## Conflict of Interest

None declared.

## Supporting information


**Figure S1.** The typical picture of PCFCCL displays a nodular (A, hematoxylin–eosin 20×) to diffuse proliferation composed of small‐ to medium‐sized centroblasts (B, hematoxylin–eosin 400×), with a variable proportion of centroblast or with a spindle cell morphology (C, hematoxylin–eosin 200×). BCL2 is usually negative (D, SABC method, 400×) and CD10 is positive (E, SABC method, 200×), whereas a residual, CD23+ positive dendritic meshwork is typically present (F, SABC method, 200×).

**Figure S2.** A representative picture of the presence of t(14;18) is depicted (A, *IGH/BCL2* Dual Color, Dual Fusion Translocation Probe, 1000×). EBV status was invariably negative (B, EBER‐ISH, 400×); slides taken from nonkeratinizing undifferentiated nasopharyngeal carcinoma were used as positive control (B, inset).

## References

[1] Swerdlow S. H., Campo E., Harris N. L., Jaffe E. S., Pileri S. A., Stein H., et al., eds. 2008 WHO classification of tumors of haematopoietic and lymphoid tissues. 4 ed IARC Press, Lyon.

[2] Zinzani, P. L. , P. Quaglino , N. Pimpinelli , E. Berti , G. Baliva , S. Rupoli , et al. 2006 Prognostic factors in primary cutaneous B cell lymphoma: the Italian Study Group for Cutaneous Lymphomas. J. Clin. Oncol. 24:1376–1382. 16492713 10.1200/JCO.2005.03.6285

[3] Senff, N. J. , J. J. Hoefnagel , P. M. Jansen , M. H. Vermeer , J. van Baarlen , W. A. Blokx , et al. 2007 Reclassification of 300 primary cutaneous B‐cell lymphomas according to the new WHOEORTC classification for cutaneous lymphomas: comparison with previous classifications and identification of prognostic markers. J. Clin. Oncol. 25:1581–1587. 17353548 10.1200/JCO.2006.09.6396

[4] Dijkman, R. , C. P. Tensen , E. S. Jordanova , J. Knijnenburg , J. J. Hoefnagel , A. A. Mulder , et al. 2006 Array‐based comparative genomic hybridization analysis reveals recurrent chromosomal alterations and prognostic parameters in primary cutaneous large B‐cell lymphoma. J. Clin. Oncol. 24:296–305. 16330669 10.1200/JCO.2005.02.0842

[5] Hoefnagel, J. J. , R. Dijkman , K. Basso , P. M. Jansen , C. Hallermann , R. Willemze , et al. 2005 Distinct types of primary cutaneous large B‐cell lymphoma identified by gene expression profiling. Blood 105:3671–3678. 15308563 10.1182/blood-2004-04-1594

[6] Pham‐Ledard, A. , M. Prochazkova‐Carlotti , L. Andrique , D. Cappellen , B. Vergier , F. Martinez , et al. 2014 Multiple genetic alterations in primary cutaneous large B‐cell lymphoma, leg type support a common lymphomagenesis with activated B‐cell‐like diffuse large B‐cell lymphoma. Mod. Pathol. 27:402–411. 24030746 10.1038/modpathol.2013.156

[7] Willemze, R. , E. S. Jaffe , G. Burg , L. Cerroni , E. Berti , S. H. Swerdlow , et al. 2005 WHO‐EORTC classification for cutaneous lymphomas. Blood 105:3768–3785. 15692063 10.1182/blood-2004-09-3502

[8] Boit Le, P. E. , Burg G., Weedon D., Sarasin A., eds. 2005 WHO classification of tumors. Pathology and genetics of skin tumors. 3 ed IARC Press, Lyon.

[9] Willemze, R. , C. J. L. M. Meijer , E. Scheffer , P. M. Kluin , W. A. Van Vloten , J. Toonstra , et al. 1987 Diffuse large cell lymphomas of follicular center cell origin presenting in the skin. A clinicopathologic and immunologic study of 16 patients. Am. J. Pathol. 126:325–333. 3548403 PMC1899577

[10] Kim, B. K. , U. Surti , A. G. Pandya , and S. H. Swerdlow . 2003 Primary and secondary cutaneous diffuse large B‐cell lymphomas: a multiparameter analysis of 25 cases including fluorescence in situ hybridization for t(14;18) translocation. Am. J. Surg. Pathol. 27:356–364. 12604892 10.1097/00000478-200303000-00009

[11] Grange, F. , T. Petrella , M. Beylot‐Barry , P. Joly , M. D'Incan , M. Delaunay , et al. 2004 Bcl‐2 protein expression is the strongest independent prognostic factor of survival in primary cutaneous large B‐cell lymphomas. Blood 103:3662–3668. 14726400 10.1182/blood-2003-08-2726

[12] Hans, C. P. , D. D. Weisenburger , T. C. Greiner , R. D. Gascoyne , J. Delabie , G. Ott , et al. 2004 Confirmation of the molecular classification of diffuse large B‐cell lymphoma by immunohistochemistry using a tissue microarray. Blood 103:275–282. 14504078 10.1182/blood-2003-05-1545

[13] Hu, S. , Z. Y. Xu‐Monette , A. Tzankov , T. Green , L. Wu , A. Belasubramanyam , et al. 2013 MYC/BCL2 protein coexpression contributes to the inferior survival of activated B‐cell subtype of diffuse large B‐cell lymphoma and demonstrates high‐risk gene expression signatures: a report from The International DLBCL Rituximab‐CHOP Consortium Program. Blood 121:4021–4031. 23449635 10.1182/blood-2012-10-460063 PMC3709650

[14] Kodama, K. , C. Massone , A. Chott , D. Metze , H. Kerl , and L. Cerroni . 2005 Primary cutaneous large B‐cell lymphomas: clinicopathologic features, classification, and prognostic factors in a large series of patients. Blood 106:2491–2497. 15947086 10.1182/blood-2005-03-1175

[15] Gulia, A. , A. Saggini , T. Wiesner , R. Fink‐Puches , Z. Argenyi , G. Ferrara , et al. 2011 Clinicopathologic features of early lesions of primary cutaneous follicle center lymphoma, diffuse type: implications for early diagnosis and treatment. J. Am. Acad. Dermatol. 65:991–1000. 21704419 10.1016/j.jaad.2010.06.059

[16] Vermeer, M. H. , F. A. Geelen , C. W., van Haselen , P. C., van Voorst Vader , M. L. Geerts , W. A., van Vloten , et al. 1996 Primary cutaneous large B‐cell lymphomas of the legs. A distinct type of cutaneous B‐cell lymphoma with an intermediate prognosis. Dutch Cutaneous Lymphoma Working Group. Arch. Dermatol. 132:1304–1308. 8915307

[17] Paulli, M. , A. Viglio , D. Vivenza , D. Capello , D. Rossi , R. Riboni , et al. 2002 Primary cutaneous large B‐cell lymphoma of the leg: histogenetic analysis of a controversial clinicopathologic entity. Hum. Pathol. 33:937–943. 12378521 10.1053/hupa.2002.126881

[18] Xie, X. , U. Sundram , Y. Natkunam , S. Kohler , Y. H. Kim , J. R. Cook , et al. 2008 Expression of HGAL in primary cutaneous large B‐cell lymphomas: evidence for germinal center derivation of primary cutaneous follicular lymphoma. Mod. Pathol. 21:653–659. 18264083 10.1038/modpathol.2008.30

[19] Wiesner, T. , B. Streubel , D. Huber , H. Kerl , A. Chott , and L. Cerroni . 2005 Genetic aberrations in primary cutaneous large B‐cell lymphoma: a fluorescence in situ hybridization study of 25 cases. Am. J. Surg. Pathol. 29:666–673. 15832092 10.1097/01.pas.0000155163.40668.e7

[20] Sehn, L. H. , and R. D. Gascoyne . 2015 Diffuse large B‐cell lymphoma: optimizing outcome in the context of clinical and biologic heterogeneity. Blood 125:22–32. 25499448 10.1182/blood-2014-05-577189

[21] Franco, R. , A. Fernández‐Vázquez , M. Mollejo , M. A. Cruz , F. I. Camacho , J. F. Garcia , et al. 2001 Cutaneous presentation of follicular lymphomas. Mod. Pathol. 14:913–919. 11557789 10.1038/modpathol.3880411

[22] Cerroni, L. , E. Arzberger , B. Pütz , G. Höfler , D. Metze , C. A. Sander , et al. 2000 Primary cutaneous follicle center cell lymphoma with follicular growth pattern. Blood 95:3922–3928. 10845929

[23] Bergman, R. , P. J. Kurtin , L. E. Gibson , P. R. Hull , T. K. Kimlinger , and A. L. Schroeter . 2001 Clinicopathologic, immunophenotypic, and molecular characterization of primary cutaneous follicular B‐ lymphoma. Arch. Dermatol. 137:432–439. 11295923

[24] Aguilera, N. S. , M. M. Tomaszewski , J. C. Moad , F. A. Bauer , J. K. Taubenberger , and S. L. Abbondanzo . 2001 Cutaneous follicle center lymphoma: a clinicopathologic study of 19 cases. Mod. Pathol. 14:828–835. 11557777 10.1038/modpathol.3880398

[25] Child, F. J. , R. Russell‐Jones , A. J. Woolford , E. Calonje , A. Photiou , G. Orchard , et al. 2001 Absence of the t(14;18) chromosomal translocation in primary cutaneous B‐cell lymphoma. Br. J. Dermatol. 144:735–744. 11298531 10.1046/j.1365-2133.2001.04128.x

[26] Lawnicki, L. C. , D. D. Weisenburger , P. Aoun , W. C. Chan , R. S. Wickert , and T. C. Greiner . 2002 The t(14;18) and bcl‐2 expression are present in a subset of primary cutaneous follicular lymphoma: association with lower grade. Am. J. Clin. Pathol. 118:765–772. 12428798 10.1309/2TJU-DNLQ-5JBA-AB4T

[27] Goodlad, J. R. , A. S. Krajewski , P. J. Batstone , P. McKay , J. M. White , E. C. Benton , et al. 2002 Primary cutaneous follicular lymphoma: a clinicopathologic and molecular study of 16 cases in support of a distinct entity. Am. J. Surg. Pathol. 26:733–741. 12023577 10.1097/00000478-200206000-00006

[28] Mirza, I. , N. Macpherson , S. Paproski , R. D. Gascoyne , B. Yang , W. G. Finn , et al. 2002 Primary cutaneous follicular lymphoma: an assessment of clinical, histopathologic, immunophenotypic, and molecular features. J. Clin. Oncol. 20:647–655. 11821444 10.1200/JCO.2002.20.3.647

[29] Vergier, B. , M. A. Belaud‐Rotureau , M. N. Benassy , M. Beylot‐Barry , P. Dubus , M. Delaunay , et al. 2004 Neoplastic cells do not carry bcl2‐JH rearrangements detected in a subset of primary cutaneous follicle center B‐cell lymphomas. Am. J. Surg. Pathol. 28:748–755. 15166666 10.1097/01.pas.0000126775.27698.6e

[30] Kim, B. K. , U. Surti , A. Pandya , J. Cohen , M. S. Rabkin , and S. H. Swerdlow . 2005 Clinicopathologic, immunophenotypic, and molecular cytogenetic fluorescence in situ hybridization analysis of primary and secondary cutaneous follicular lymphomas. Am. J. Surg. Pathol. 29:69–82. 15613857 10.1097/01.pas.0000146015.22624.c7

[31] Streubel, B. , B. Scheucher , J. Valencak , D. Huber , P. Petzelbauer , F. Trautinger , et al. 2006 Molecular cytogenetic evidence of t(14;18)(IGH;BCL2) in a substantial proportion of primary cutaneous follicle center lymphomas. Am. J. Surg. Pathol. 30:529–536. 16625101 10.1097/00000478-200604000-00015

[32] Abdul‐Wahab, A. , S. Y. Tang , A. Robson , S. Morris , N. Agar , E. M. Wain , et al. 2014 Chromosomal anomalies in primary cutaneous follicle center cell lymphoma do not portend a poor prognosis. J. Am. Acad. Dermatol. 70:1010–1020. 24679486 10.1016/j.jaad.2014.01.862

[33] Pharm Ledard, A. , A. Cowppli‐Bony , A. Doussau , M. Prochazkova‐Carlotti , E. Laharanne , T. Jouary , et al. 2015 Diagnostic and prognostic value of BCL2 rearrangement in 53 patients with follicular lymphoma presenting as primary skin lesions. Am. J. Clin. Pathol. 143:362–373. 25696794 10.1309/AJCP4SUBR4NPSPTN

[34] Adam, P. , R. Baumann , J. Schmidt , S. Bettio , K. Weisel , I. Bonzheim , et al. 2013 The BCL2 E17 and SP66 antibodies discriminate 2 immunophenotypically and genetically distinct subgroups of conventionally BCL2‐”negative” grade 1/2 follicular lymphomas. Hum. Pathol. 44:1817–1826. 23642737 10.1016/j.humpath.2013.02.004

