# Supplementary figures and images for: Primary cutaneous B‐cell lymphoma other than marginal zone: clinicopathologic analysis of 161 cases: Comparison with current classification and definition of prognostic markers
Source: Cancer Med. 2016 Sep 26;5(10):2740–55. doi: 10.1002/cam4.865 (PMC5083727; doi:10.1002/cam4.865)

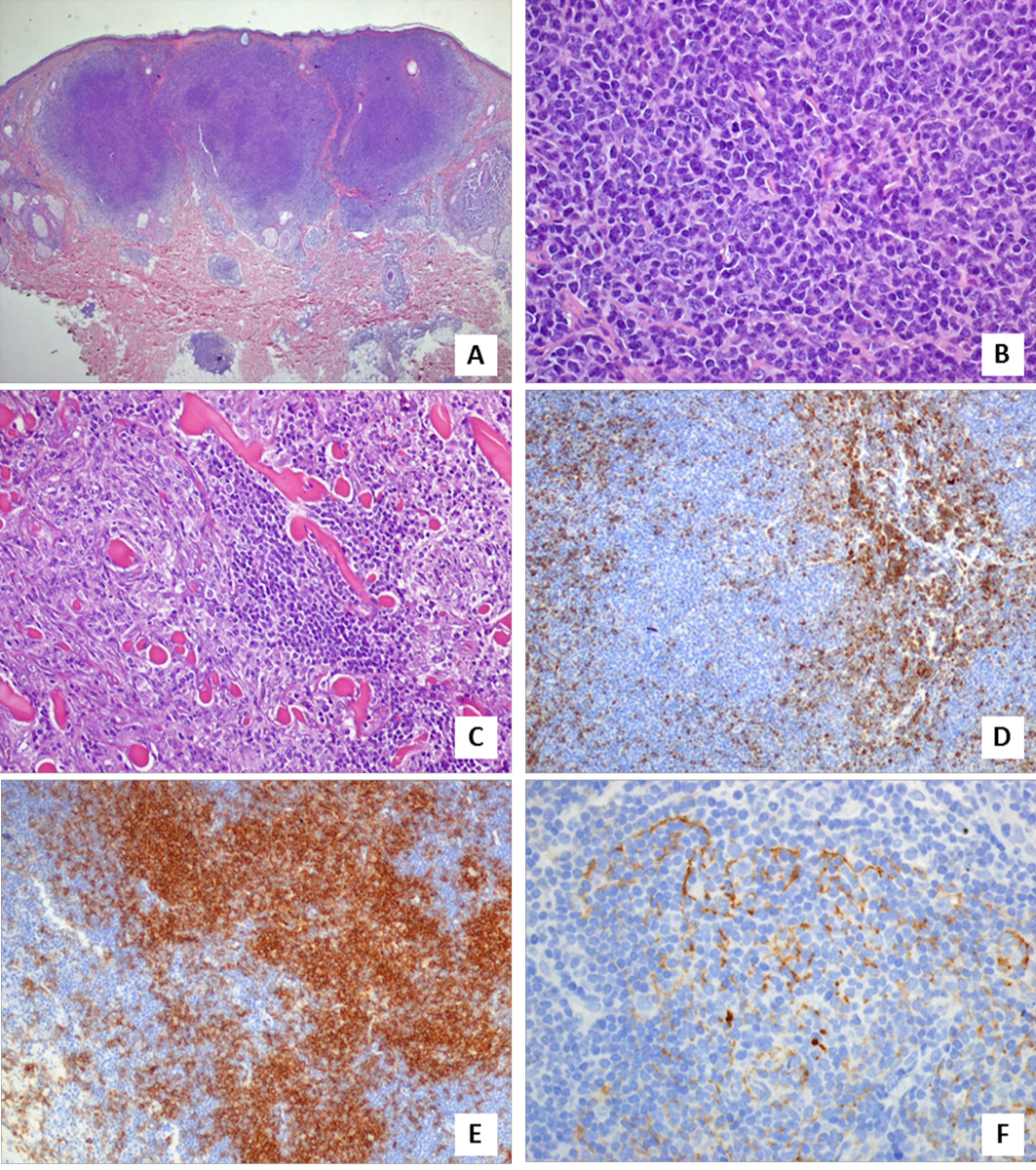

Supplement: Supplementary file 1 — Figure S1. The typical picture of PCFCCL displays a nodular (A, hematoxylin–eosin 20×) to diffuse proliferation composed of small‐ to medium‐sized centroblasts (B, hematoxylin–eosin 400×), with a variable proportion of centroblast or with a spindle cell morphology (C, hematoxylin–eosin 200×). BCL2 is usually negative (D, SABC method, 400×) and CD10 is positive (E, SABC method, 200×), whereas a residual, CD23+ positive dendritic meshwork is typically present (F, SABC method, 200×). [file CAM4-5-2740-s001.tiff]

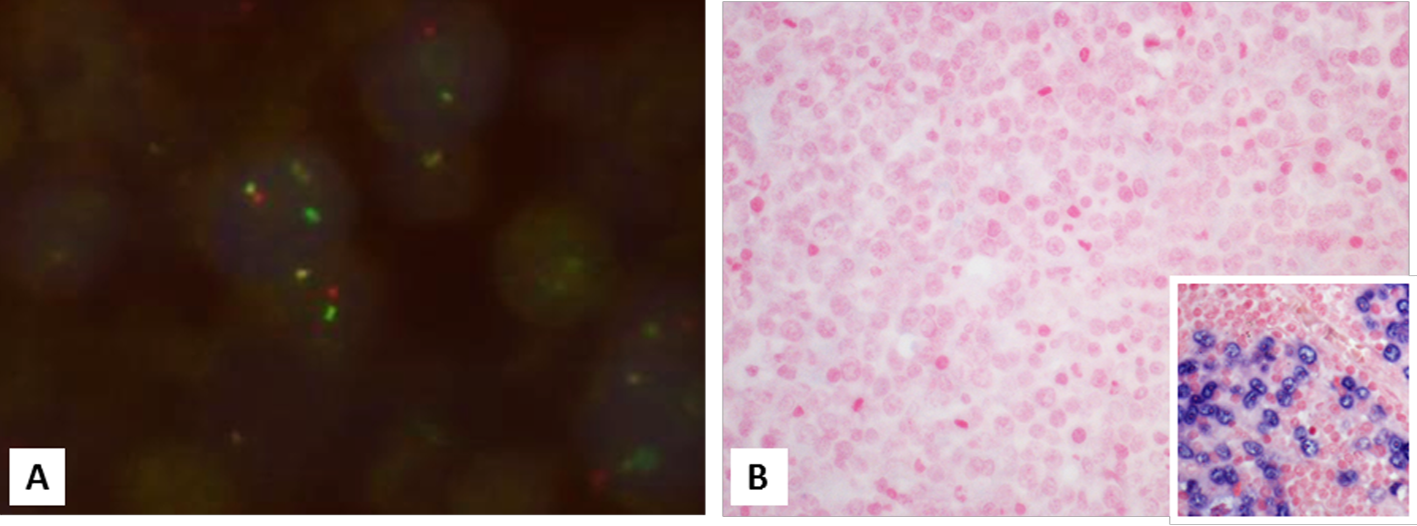

Supplement: Supplementary file 2 — Figure S2. A representative picture of the presence of t(14;18) is depicted (A, IGH/BCL2 Dual Color, Dual Fusion Translocation Probe, 1000×). EBV status was invariably negative (B, EBER‐ISH, 400×); slides taken from nonkeratinizing undifferentiated nasopharyngeal carcinoma were used as positive control (B, inset). [file CAM4-5-2740-s002.tiff]
